# Pharmacological attributes of *Bacopa monnieri* extract: Current updates and clinical manifestation

**DOI:** 10.3389/fnut.2022.972379

**Published:** 2022-08-18

**Authors:** Urooj Fatima, Sonam Roy, Shahnawaz Ahmad, Sabeeha Ali, Wafaa M. Elkady, Ilyas Khan, Rana M. Alsaffar, Mohd Adnan, Asimul Islam, Md. Imtaiyaz Hassan

**Affiliations:** ^1^Centre for Interdisciplinary Research in Basic Sciences, Jamia Millia Islamia, New Delhi, India; ^2^School of Biotechnology, IFTM University, Moradabad, India; ^3^Department of Pharmacognosy and Medicinal Plants, Faculty of Pharmacy, Future University in Egypt, New Cairo, Egypt; ^4^Department of Mathematics, College of Science Al-Zulfi, Majmaah University, Al-Majmaah, Saudi Arabia; ^5^Department of Pharmacology and Toxicology, College of Pharmacy, Prince Sattam Bin Abdulaziz University, Al-Kharj, Saudi Arabia; ^6^Department of Biology, College of Science, University of Hail, Hail, Saudi Arabia

**Keywords:** pharmacological potential, tau aggregates, *Bacopa monnieri*, anti-cancer agents, neurodegenerative diseases

## Abstract

*Bacopa monnieri* has been used for centuries in Ayurvedic medicine, alone or in combination with other herbs, as a memory and learning enhancer, sedative, and anti-epileptic. This review aimed to highlight the health benefits of *B. monnieri* extracts (BME), focusing on anti-cancer and neurodegenerative diseases. We examined the clinical studies on phytochemistry and pharmacological application of BME. We further highlighted the mechanism of action of these extracts in varying types of cancer and their therapeutic implications. In addition, we investigated the underlying molecular mechanism in therapeutic interventions, toxicities, safety concerns and synergistic potential in cognition and neuroprotection. Overall, this review provides deeper insights into the therapeutic implications of Brahmi as a lead formulation for treating neurological disorders and exerting cognitive-enhancing effects.

## Introduction

**Figure 1:**
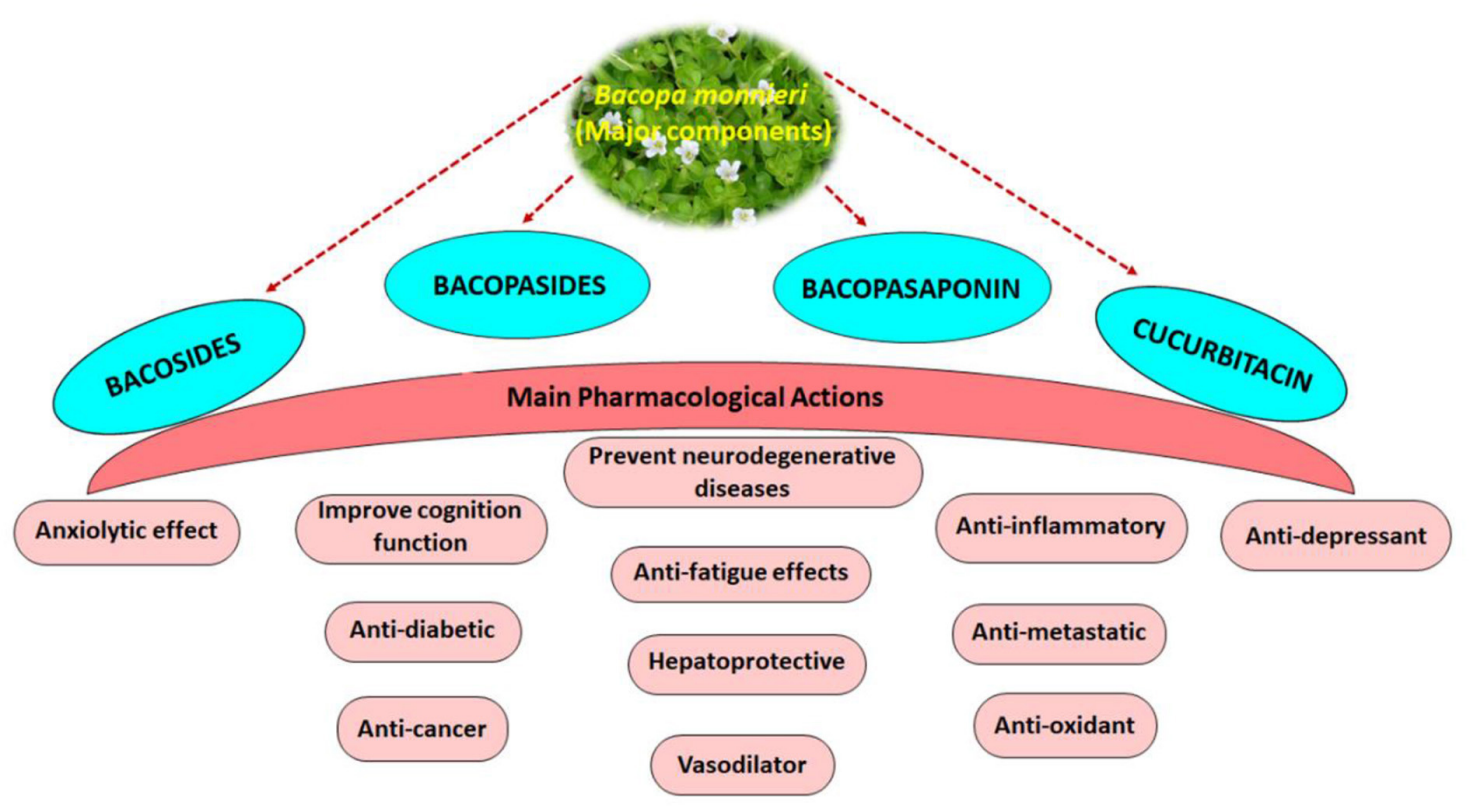
An overview of the pharmacological effects of major bioactive constituents of *Bacopa monnieri*.

*Bacopa monnieri* (Brahmi) is a well-known perennial, creeping herb possessing bioactive formulation in the Indian Ayurveda system, implicated in the therapeutic management of numerous diseases. This herb was used by Ancient Vedic scholars due to its pharmacological effect, especially as a nerve tonic and nootropic booster. However, to better understand *Bacopa monnieri's* role in several neurological disorders and memory-related diseases, it is necessary to understand its active phytochemical constituents and underlying mechanism of action. Bioactive components of Brahmi belong to alkaloids, saponins, flavonoids, triterpenes and cucurbitacin, having potential role in neuroprotection (Figure 1).

Among various medicinal Ayurvedic herbs, *Bacopa monnieri* is considered a herb of grace and commonly known as Brahmi, belonging to the family Scrophulariaceae. It is a small creeping herb with numerous branches, small oblong leaves, and light purple flowers found throughout the Indian subcontinent in wet, damp, and marshy area (1). Brahmi has had a rich historical and religious background for more than 1,400 years. It has been reported for various pharmacological activities. Brahmi is used as a brain booster by amassing the information evolved through experience over years and years because it acts as a rejuvenator for the brain and nervous system.

The extracts of *B. monnieri* are well-recognized for their antioxidant activity with numerous modes of action to protect the brain against oxidative damage and cognitive decline in the elderly. The cognition-promoting roles of *B monnieri* can be due to the antioxidant effects of alcoholic extracts and bacoside (2). Based on animal study results, the *B. monnieri* extract and bacosides were shown to enhance antioxidant status in the brain region of the hippocampus, frontal cortex, and striatum (3). In the diabetic model, it ameliorates diabetes-induced-oxidative stress (4). The evidence suggests that *in vivo* chronic cigarette smoke exposure enhances oxidative stress, and bacoside A was found to protect against cigarette smoking-induced cerebrovascular diseases by decreasing the formation of free radicals through its antioxidant potential (5).

Earlier research also reported a dose-dependent free radical scavenging ability and a protective effect of methanolic extract *B. monnieri* against DNA (6, 7). *In vitro* and *in vivo* studies in *C. elegans* done by Phulara et al. (8) provide evidence that *B monnieri* has antioxidant activity and is capable of up-regulating the expression of the gene hsp-16.2 associated with stress tolerance, which greatly improves the lifetime of *C. elegans* under stress conditions (8).

The nephroprotective efficacy of *B. monnieri* in mice against Tracolimus-induced kidney toxicity has been reported (9). This protective efficacy is accompanied by a significant attenuation of oxidative stress and maybe through free radical scavenging activity *of B. monnieri*. This evidence suggests that *B. monnieri* might have potential as an adjunct therapy in which free radical production plays a vital role and would be useful in advancing novel *B. monnieri* herbal drugs for various stress-related human complications.

The extract of Brahmi and its isolated valuable therapeutic agents have been extensively investigated for their nootropic effects, antioxidant, antimicrobial properties and analgesic activity, etc. These traditional pharmacological claims have been bolstered by large-scale research and clinical studies (10). Brahmi has been the focus of research as a versatile therapeutic agent for various disorders and neurodegenerative diseases.

This review article addresses the major phytochemical profile and pharmacological attributes emphasizing the neuroprotective role of *Bacopa monnieri*. We further highlighted the underlying biochemical mechanisms of action of *Bacopa monnieri* in neuroprotection and other diseases.

### Bioactive constituents and their functional significance

**Figure 2:**
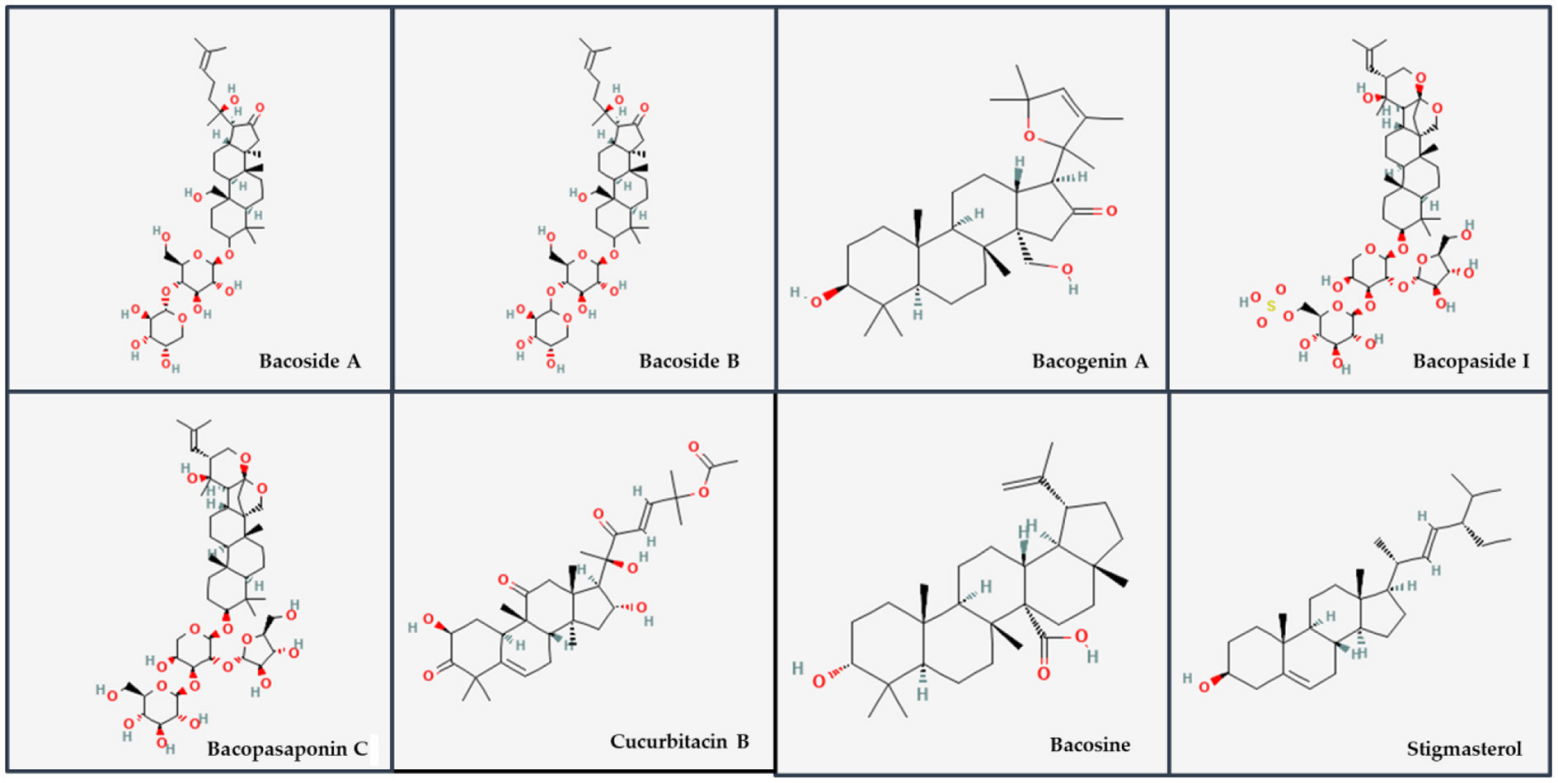
Representative structures of major phytoconstituents present in extracts of Bacopa monnieri. Structures were downloaded from PubChem (https://pubchem.ncbi.nlm.nih.gov) with their corresponding PubChem CIDs, Bacoside A (92043183), Bacoside B (121596009), Bacogenin A (101600046), Bacopaside I (21599442), Bacopasaponin C (21599443), Cucurbitacin B (5281316), Bacosine (71312547), and Stigmasterol (5280794).

*Bacopa monnieri* plant is rich in clinically critical secondary metabolites such as saponins, alcohols, steroids, alkaloids, glycosides, sterol glycosides, phenylethanoid glycosides, sugars, amino acids, flavonoids and cucurbitacins (11–13). In addition, Brahmin, Hydrocotyline, Nicotine, Herpestine, D-mannitol, stigmasterol, glutamic acid, aspartic acid, alanine, and serine are specific amino acids are present in the extracts of *Bacopa monnieri*. Structural features of different components of *Bacopa monnieri* extracts are illustrated in Figure 2. The major component are saponins comprising bacosides, bacopasides (13), Bacosaponins (14, 15), Betulinic acid, etc.

Bacosides are significant components of *Bacopa monnieri and* play essential roles in neuronal health. Structurally, bacoside-A (PubChem ID: 92043183) is an amphiphilic chemical compound containing both sterol and sugar moieties. Deepak et al. (16) identified and characterized 12 analogs of the bacosides, known as bacopasides I-XII. Most of the glycosides have sugar chains attached to the C-3 only (classified as monodesmosides) and in few to both C-3 and C-20 (classified as bidesmosides) of the aglycone unit (17). They protected the cytotoxicity and DNA damage of neurons implicated in Alzheimer's disease (AD) and repaired the impaired neurons by enhancing kinase activity and neuronal synthesis (18). Bacosides A and B are responsible for most neuropharmacological and nootropic effects (14, 16). Bacoside A contains four saponin glycosides viz. bacopaside II, bacopaside X, bacoside A3 and bacopasaponin C (16). In contrast, bacoside B only varies in optical rotation with bacoside A and consists of bacopaside IV, V, N1, & N2 (16, 19, 20). Bacoside A is pharmacologically more active than bacoside B.

Bacogenin A1–A5 is the acid hydrolyzed derivatives of bacosides (21–23), and among which ebelin lactone (bacogenin A4) (24) is the major component (25). Ramasamy and co-workers suggested that ebelin lactone and bacogenin A1 bind highly to CNS receptors. Bacopasides I-XII are important saponins that interact with sterols and are involved in membrane disruption (13, 26–28).

Other jujubogenin and pseudojujubogenin derivatives whose role is yet to be explored are termed Bacopasaponin A-H (29, 30). Among these, bacopasaponin C comprises 0.3–0.6% of the ethanolic extracts of BM (31). It is a glycosidic pseudojujobogenin with glucose and rhamnose as sugar units (28). This terminal glucose moiety is self-targeting toward cells with their specific receptors and is responsible for the antileishmanial property of bacopasaponin C (32).

Cucurbitacin displays varied types of biological properties in plants and animals. Four cucurbitacins, bacitracin A-D and cucurbitacin E, isolated from the ethanolic extract of the dichloromethane (DCM) portion of the BM plant (33). Betulinic acid and its derivative dihydro betulinic acid (IC_50_ = 0.5 μM) are the most potent pentacyclic triterpenoid inhibitor of eukaryotic topoisomerase I for anti-cancer drug designing (34). In addition, bacosine protected against oxidative damage in alloxanized diabetes and increased peripheral glucose consumption. Bacosine administration also upturned weight loss in diabetic rats and prohibited *in vitro* glycosylation of hemoglobin (35).

### Cancer prevention

*B. monnieri* has an anti-cancer efficacy on different types of cancer. Palethorpe et al. (36) reported that bacopaside I and bacopaside II, a terpenoid from *B. monnieri*, can synergistically block the functional activity of the membrane transport system aquaporin, AQP1 is also reported to contribute to tumor progression. The reduced transcriptional expression of AQP1 inhibits proliferation, migration and invasion in breast cancer cell lines.

Similarly, Pei et al. (37) reported that bacopaside I and bacopaside II blocked AQP1 and inhibited colon cancer cell lines (37). This work is further supported by Smith et al. (38). Bacopaside II has been shown to activate autophagy by inhibiting G2/M cell cycle transition and inducing apoptosis of low and high AQP1-expressing colon cancer cells.

Based on these findings, bacopasides have been proposed as potential novel lead compounds for the pharmaceutical production of selective AQP blockers in cancer treatment. Hepatocellular carcinoma (HCC) represents the fifth most common cancer globally and is related to mortality worldwide (39). *B. monnieri's* alcohol extract has been highlighted as an effective antioxidant, free radical scavenger, and a potential anti-lipid peroxidative agent (3, 40–42).

Janani et al. (43) showed that BM extract, Bacoside A, can prevent N-nitrosodiethylamine (DEN)-induced hepatoma by inhibiting lipid peroxidation and by enhancing the levels of antioxidant enzymes in Wistar albino rat. In another study, Janani et al. **(author?)** (44) investigated the effect of Bacoside A on the activities and expression of matrix metalloproteases (MMPs) enzymes, i.e., MMP-2 and MMP-9 in DEN-induced HCC. They reported that Bacoside A employs its anti-metastatic effect against DEN-induced HCC by suppressing the activities and expressions of MMP-2 and MMP-9 enzymes responsible for metastasis in various tumors (44).

Nitrobenzene is a hazardous air pollutant and is considered a human carcinogen that affects the liver, brain, blood, and stomach. The ethanolic extract of BM at the dose of 200 mg/kg showed a good hepatoprotective effect in nitrobenzene-induced liver damage in mice model by an increase in SOD, CAT and GPx enzymes and by normalizing the serum marker enzymes such as aspartate transaminase, alanine transaminase, and alkaline phosphatase. In contrast, the levels of these serum marker enzymes increased in the carcinogen-administered mice models (45).

Another common and aggressive tumor that causes the highest deaths worldwide is glioblastoma (GBM). A glioblastoma is a brain tumor with an inferior prognosis that is highly vascularized, infiltrative, and exacerbates its tumor potential. All these features are therapeutic objectives in glioblastoma treatment, including surgical removal accompanied by chemotherapy and radiotherapy (46, 47). Existing therapies have not adequately handled the patient, so classical therapies have had to expand and integrate new alternative approaches, like natural compounds. Other targets in the treatment of glioblastoma are the inhibition of the notch signaling pathway that contributes to a decrease in glioblastoma cell proliferation and self-renewal (48, 49), the receptor for an epidermal growth factor (EGFR) (50), nuclear factor kappa-light-chain-enhancer of activated B cells (NF-κB) (51). Natural substances are emerging as potential therapies to address GBM growth (52–55). A previous study also documented the apoptotic activity of bacoside A in brain tumor cells, GBM (56).

Neuroblastoma is an embryonic cancer of the autonomic nervous system arising from the embryonic sympathoadrenal lineage of the neural crest. For children between 1 and 5 years of age, neuroblastoma is the main cause of death from pediatric cancer and accounts for around 13% of pediatric cancer mortality (57). The neuroprotective potential of *B. monnieri* has been studied with its active ingredients, such as Bacopasaponins, Betulinic acid, Bacoside A and B, etc. Thus, researchers have taken advantage of the neuroprotective property of *B. monnieri* in searching for natural remedies for this pediatric cancer.

Several studies reported that the extract of this herbal plant prevents hydrogen peroxide-induced oxidative damage in human neuroblastoma cell lines (58, 59). These findings suggested that *B. monnieri* effectively treats different forms of cancer and can shield against brain damage, and improve brain development.

### Management of diabetes nephropathy

Diabetes mellitus (DM) is a chronic metabolic condition with life-threatening complications characterized by hyperglycemia, hyperlipidemia, hyper aminoacidemia, and hyperinsulinemia. According to the seventh edition of the World Diabetes Atlas released by the International Diabetes Federation (IDF), as of 2015, about 415 million people worldwide live with diabetes. This number will likely increase to 642 million by 2040 (60). Drugs are available to monitor and treat diabetic patients, but complete diabetes recovery has not been reported.

Alternative to these drugs, plants provide potential antidiabetic effects and are commonly used in many conventional medicine schemes to prevent diabetes (61–64). In a study conducted by Gosh et al., ethanolic extract of the aerial parts of *B. monnieri* was evaluated against antioxidant and antihyperglycemic activity in the Wistar mice model and elucidated that BME prevents significant elevation of glycosylated hemoglobin *in vitro* with IC_50_ value being 11.25 μg/ml that is comparable with the control drug α-tocopherol (65). This IC_50_ value reached 7.44 μg/mL when treated with only Bacosine, a triterpene from *B. monnieri* (35).

The previous study has also indicated that an isolate of BM, stigmasterol is effective in streptozotocin-nicotinamide-induced diabetic nephropathy (DN), i.e., it inhibits the progression of chronic complications of diabetes *via* reducing the formation of advanced glycation end products and amelioration of oxidative stress (66). Moreover, the previous research demonstrated that *B. monnieri* reduced serum glucose and increased diabetic rat body weight (4, 67).

### Parkinson's disease

Parkinson's disease (PD) is a slowly progressive, degenerative disorder characterized by degeneration of nerve cells in the substantia nigra region and aggregation of a key protein, alpha-synuclein, in the striatum and adjacent brain regions. *B. monnieri* modulates PD (68, 69). Evidence from the animal model showed the anti-Parkinson's effect of *B. monnieri* extract. Bacosides, an alcoholic extract of *B. monnieri*, was explored in a *Caenorhabditis elegans* model where it exhibited decreased aggregation of α-synuclein and prevented dopaminergic neurodegeneration, restoring nematode lipid material (70). Another group of researchers reported that Bacopa treatment to the MPTPP (1-methyl-4-phenyl-1,2,3,6-tetrahydropyridine) induced parkinsonian mice model offers nigrostriatal dopaminergic neuroprotection against MPTP-induced parkinsonism by modulating the behavioral effects of oxidative stress and apoptotic machinery (71).

Moreover, the effect of the extract of *B. monnieri* leaf was studied on transgenic model flies of *Drosophila* that expressed normal human alpha-synuclein in their neurons. The extract improves behavioral defects and decreases oxidative stress and apoptosis in the flies of PD model brains (72). All these findings confirm the efficacy of *B. monnieri* as a novel therapeutic for PD treatment.

### Alzheimer's disease

A significant and growing public health concern is AD. It is associated with cognitive impairment and dementia and is characterized by the accumulation of amyloid-β peptides in senile plaques and abnormally phosphorylated tau proteins (73–75). The root cause of a group of neurodegenerative disorders collectively known as “tauopathies” was confirmed to be hyperphosphorylation of tau protein. The detrimental effect is the loss of affinity between this protein and the microtubules, increased fibrillary aggregate production, and accumulated insoluble neurofibrillary tangles (76–78).

**Figure 3:**
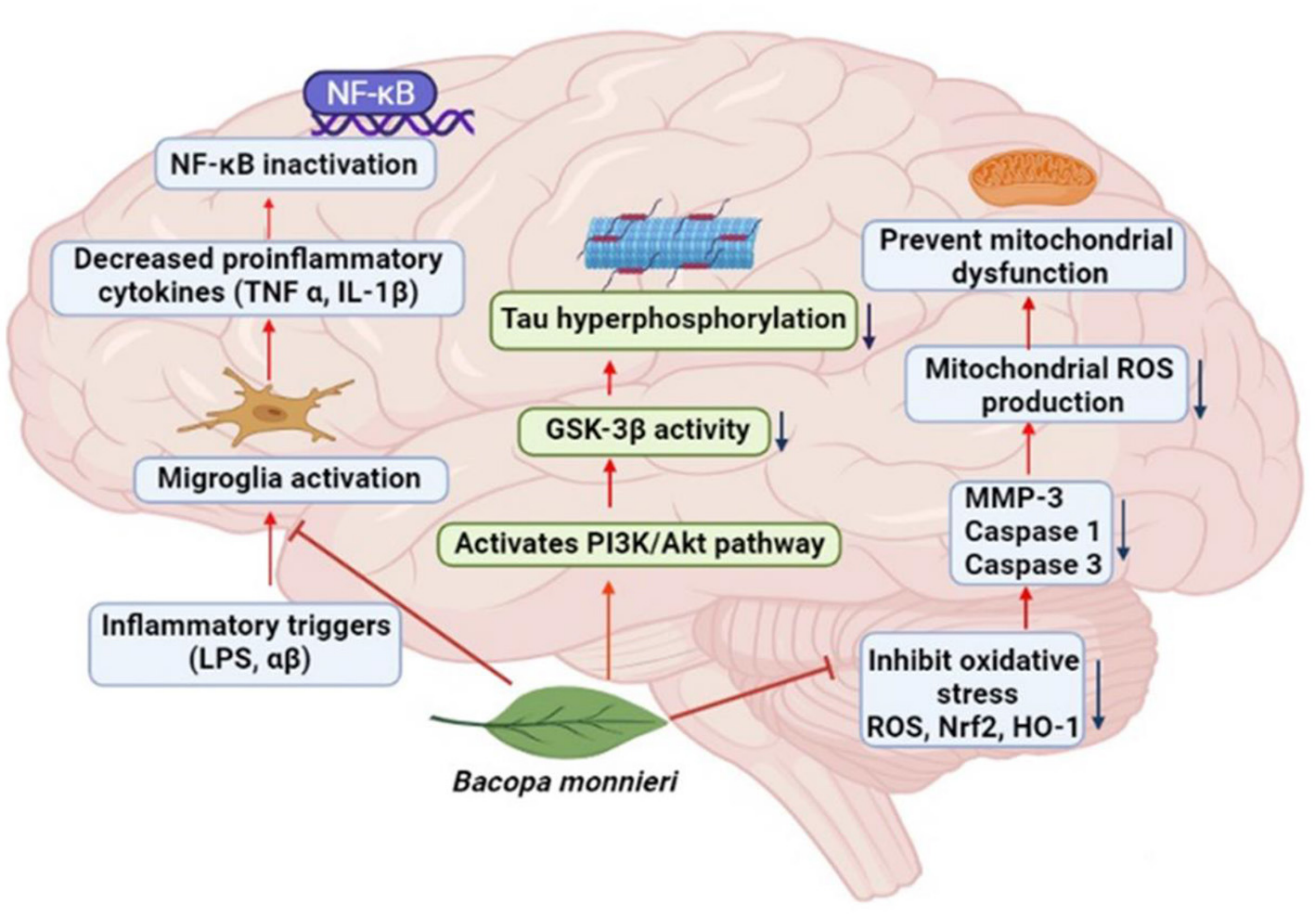
The action mechanism of *Bacopa monnieri* against neurodegenerative diseases.

In animal models of AD, *Bacopa* has been shown to suppress beta-amyloid deposits in the brain (79). This herb showed a significant memory-enhancing influence. Research demonstrated that this herb facilitates acquisition, retention, and recovery (80). Evidence also showed that many herbs effective against AD (81–85). Brahmi and its active component have exclusively been explored in AD treatment among all herbs. Recently, it has been reported that alcoholic extract of *B. monnieri* significantly improves escape latency time in the Morris water maze test and facilitates the reduction of neurons and cholinergic neuron densities in a rat model of AD (86). A detailed mechanism of action of *B. monnieri* in neurodegenerative disease is described in Figure 3.

Holcomb et al. (87) studied the PSAPP rodent model. They revealed that the administration of *Bacopa extract* to mice expressing APP and PSEN-1 mutation diminished amyloidogenic proteins Aβ40 and Aβ42 levels in the brain by ~60%. *In vitro* study explored by Mathew et al. (88) on anti-amyloidogenic potential found that *Bacopa* almost completely reduced the development of amyloid fibrils and greatly separated the preformed amyloid fibrils.

An exciting *in silico* study showed that Bacopasaponin G and Bacopasaponin N2, two saponins from *B. monnieri*, might be effective in AD therapy. Compared with Donepezil, these two saponins exert a more favorable binding affinity with the Caspase-3 and tau-protein kinase I (TPK I) receptors, therapeutic targets in AD (89). These findings indicate that this herb is a potential cognitive enhancer and promises to be a novel agent in AD.

### Pre-clinical studies

Preclinical testing was done to validate the effectiveness of *B. monnieri* extract as a herbal medicinal drug. According to several *in vitro* and *in vivo* investigations, BME appears to be helpful in the treatment/prevention of neurodegenerative diseases and other age-related disorders.

#### *In vitro* studies

Limpeanchob et al. (90) evaluated the neuroprotective effect of *B. monnieri* extracts by assessing the viability of cultures of primary cortical neuron cells treated with 50 μM aggregated Aβ 25–35 in the presence and absence of BME. In the presence of 100 μg/ml of BME, the survival rate of the cultured cells increases while the survival rate reduces in the absence of BME.

Neuronal death induced by amyloid peptide exhibited a high 2-fold rise in acetylcholine esterase (AChE) concentration, while those treated with Brahmi extract had a near-normal concentration of AChE. This study validates that Brahmi extract increases the neuronal survival rate of neuronal cells by suppressing AChE activity. Likewise, BME pre-treatment significantly reduced scopolamine-induced PC12 cell death, and viability was restored at 85.75% of control with 100 g/mL extract of BM. Pre-treatment with BME reduced the release of lactate dehydrogenase (LDH) by up to 22.42% of the total, compared to 30% in the scopolamine-treated group. BME also improved scopolamine effects by decreasing AChE and increasing muscarinic-1 receptor and BDNF expression (91).

Another study conducted by Malishev et al. (92) in the SH-SY5Y cell line demonstrated that Bacoside A at 50 M significantly inhibited cytotoxicity, fibrillation, and, in particular, membrane interactions of A (1–42) (A42). Bhatia et al. (93) evaluated the protective effect of BME against hydrogen peroxide (H_2_O_2_) induced oxidative damage in a cellular model of neuroblastoma IMR32 cells. These protective effects possibly were associated with an increase in glutathione levels, enhancing endogenous defense machinery and maintaining membrane integrity. To better characterize the neuroprotective effect of BME at the molecular level, RT-PCR and immunofluorescence were used to observe the expression of NF200 (an intermediate filament in neurons) and heat shock proteins (HSP70 and mortalin). In normal conditions, NF200, HSP70 and mortalin are assigned for cytoarchitecture and axonal transport, proper functioning of the cell under normal and stress conditions and cell proliferation, respectively. The elevated expression of these stress markers because of H_2_O_2_-induced oxidative stress causes brain injury, and varied pathological conditions, including cerebral ischemia and neurodegenerative diseases. The expression of these three oxidative stress markers was significantly alleviated after the pre-treatment of BME, which supports the neuroprotective effect of BME.

Glioblastoma multiforme (GBM) is the most aggressive malignant brain tumor, with a high proliferative rate and invasiveness. Notch1 signaling has been associated with anti-apoptotic behavior in various cellular contexts. Notch1 receptor promotes glioblastoma cells' survival by regulating the anti-apoptotic Mcl-1 protein. Inhibition of Notch1 signaling through knockdown of notch receptors sensitizes glioblastoma cells for anti-tumor treatment. The notch signaling pathway has thus proved to be a novel therapeutic target in treating GBM (94). Introduction of Bacoside A in human glioblastoma cell line U-87 MG causes cell cycle arrest and apoptosis by inhibiting the Notch1 receptors and sensitizes glioblastoma cells to apoptosis (95).

#### *In vivo* studies

*B. monnieri* as a neuroprotective agent was evaluated by *in vivo* studies using various experimental models. *In vivo* study demonstrated in *Mus musculus* (house mouse) that D-Galactose and Sodium nitrite induced impaired cognitive functions that were significantly ameliorated by administering BME (100 mg/kg of body weight), confirming that BME has anti-Alzheimer's properties (96). The cholinergic and glutamatergic networks and their interactions are involved in cognitive dysfunction associated with AD. The glutamatergic system is essential in regulating synaptic plasticity and cognition (97, 98).

Administration of *B. monnieri* ameliorates olfactory bulbectomy (OBX) induced cognition dysfunction in mouse models by the protection of cholinergic systems and by activating the synaptic proteins to induce synaptic plasticity (99). Administration of BME was found to facilitate the scopolamine effect by downregulating cholinesterase (ChE) in albino mice which was observed by improved performance on the Morris Water Maze Test. In this study, BME treatment showed a significant increase in step-down latency (SDL), which may prove to be a reliable memory restorative agent in curing dementia seen in AD (100). Gamma-aminobutyric acid (GABA) and brain-derived neurotrophic factor (BDNF) are well-known to be related to synapse recovery, neuronal survival, and neuronal protection. Thus, GABAergic transmission is thought to play a role in neurodegenerative diseases' etiology and regenerative processes by maintaining equivalent neurotransmission in the CNS.

Therefore, GABAergic transmission stability may be a therapeutic solution in many neurodegenerative disorders. Piyabhan et al. (101) demonstrated the effects of Brahmi in a PCP-induced schizophrenic-like model, including partial restoration of cognitive deficit and neuroprotection. They elucidated its underlying mechanism of action by increasing GABAergic neurons. Singh et al. (102) investigated the effect of BME on MPTP-induced nigrostriatal dopaminergic neurodegeneration in mice.

Their study demonstrated that BME has neuroprotective and neurorescuive effects. The overall *in vitro* and *in vivo* studies suggested that BME can mitigate memory impairment and neurodegenerative disorders. Table 1 outlines the specific impact of *B. monnieri* extract on various study designs (*in vitro and in vivo*) of neurodegenerative diseases.

**Table 1:** Summary of *in vitro* and *in vivo* studies.

**S. No**.	**Cell line/Model**	**Cytotoxicity induced/culture prepared**	**Protein/pathway involved**	**Dosage and effect of BME treatment**	**Mechanism of action**	**References**
***In vitro*** **study**						
1.	Primary cortical cultured neurons	Aβ25–35 (50 μM)	β-amyloid	100 g/mL protect neurons from beta-amyloid-induced cell death	Inhibitory effect on amyloid peptide-activated intracellular AChE activity.	(90)
2.	PC12	Scopolamine (3 μg/ml)	BDNF, MUS-1 and AChE	100 μg/mL of BME ameliorated the mitochondria and plasma membrane damage	Down-regulation of AChE. Up-regulation of BDNF receptor expression. Up-regulation of muscarnic-1 receptor expression.	(91)
3.	SH-SY5Y	Aβ42 (10 μM)	β-amyloid	50 μM of Bacoside-A inhibits βamyloid cytotoxicity, fibrillation, and membrane interactions.	Prevent self-assembly of oligomers.	(92)
4.	IMR32	H_2_O_2_ (250 μmol·L−1)	NF200, HSP70, and mortalin	Below 100 μg·mL−1 prevent oxidative damage	By downregulating the NF200 expression of IMR32, HSP70, and mortalin cells.	(93)
5.	U-87 MG	U-87 MG in DMEM supplemented with 10% FBS	Notch signaling pathway	80 μg/mL of Bacoside A Induced Sub-G0 Arrest and Early Apoptosis	Induced cell death and apoptosis.	(95)
***In vivo*** **study**						
6.	*Mus musculus*	D-Galactose and Sodium nitrite	ATPase system	100 mg/kg body weight of BME	Inhibition of calcium-ion influx into cell membranes.	(96)
7.	Male ddY mice	OBX mice	Glutamatergic and Cholinergic systems	50 mg/kg of BME	Facilitation cholinergic neurotransmission. Modulation Hippocampal synaptic plasticity. Mobilization of intracellular Ca^2+^ ion.	(99)
8.	Albino mice	scopolamine (40 mg/kg i.p.)	Cholinergic system	100 mg/kg of BME	Inhibition of acetylcholinesterase. Activation of choline acetyltransferase.	(100)
9.	Male Wistar rats	PCP (2 mg/kg)	Calcium-binding proteins (CB, PV, CR) localize mainly in GABAergic neurons.	225 mg of BME	By restoration of GABAergic neurons.	(101)
10.	Swiss Albino mice	MPTP (30 mg/kg BW)	Dopamine degradation pathway	40 mg/kg BW	By maintain dopamine concentrations either by increasing dopamine synthesis or by inhibiting dopamine degradation.	(102)

*DMEM, Dulbecco's Modified Eagle Medium; FBS, Fetal Bovine Serum; OBX, Olfactory Bulbectomized; PCP, Phencyclidine (1-(1-phenylcyclohexyl) piperidine); MPTP, 1-methyl-4-phenyl-1,2,3,6-tetrahydropyridine; CB, Calbindin; PV, Parvalbumin; CR, Calretinin*.

### *B. monnieri* extracts in clinical trials

Manifold clinical studies provide evidence in the form of placebo-controlled, randomized, and double-blind trials to support the cognitive benefits of *B. monnieri* supplementation. The potential of this ayurvedic medicine as a dietary supplement for prevention or as a candidate drug to cure acute and chronic neurodegenerative conditions seems relevant. Accordingly, various clinical trials evaluated their efficacy in mental aging neuropsychiatric diseases.

To investigate the effects of BME (KeenMind) on cognitive performance in healthy adults with an age spectrum between 18 and 60 years, Stough et al. (103) used a double-blind placebo-controlled trial and a series of well-validated neuropsychological assessments. A total of 46 participants were randomly allocated to 1 of 2 treatment conditions. Capsules of 320 mg of BME were given for 12 weeks which significantly improved verbal learning, early information processing, and memory strengthening in participants compared to non-treated groups. The authors support the previously published studies that chronic dosages of *B. monnieri* (KeenMind) for 90 days improve accuracy in more complex cognitive tasks (104). A significant difference in the finding of the later study from those of the earlier study (103) was the lack of reduction in state anxiety and involvement of speeded computerized tasks in the present study. Also, Roodenrys et al. (105) studied the effects of 90 days of BM (KeenMind) supplementation on memory-enhancing out-turn in 76 volunteers with an age spectrum between 40 and 65 years.

Another randomized, placebo-controlled, double-blind clinical study was performed by Morgan and Stevens (106) to see the effect of Brahmi (BacoMind) in improving older adults' memory performance. They all used the same dosage amount to see the cognitive impact of BME (KeenMind). Their findings revealed that Brahmi (BacoMind) could be used as a memory enhancer. Peth Nui et al. (107) had evaluated the effect of 300 mg of Brahmi on cognitive processing, attention, working memory, cholinergic and monoaminergic functions in 60 healthy adults. Another group of researchers, in their double-blind, placebo-controlled clinical trial of 320 mg and 640 mg doses involving 17 healthy adults (aged between 18 and 44 years), reported the acute effects of Brahmi (CDRI 08) on stress and mood swings caused by multitasking (108).

In another study, the randomized, double-blind placebo-controlled efficacy of *B. monnieri* extract (CDRI 08) enhanced cognitive performances. Here, 100 subjects aged between 6 and 14 were given 1 × 160 mg capsule of either Bacopa or placebo if weight is between 20 and 35 kg or 2 × 160 mg capsules of either Bacopa or placebo per day weight is above 35 kg for 16 weeks. This study demonstrated that BM is significantly beneficial for the symptoms of hyperactivity or attention deficit hyperactivity disorder (ADHD) and suggestive of cognitive improvement (109). Anhedonia (the reduced ability to experience pleasure) is a hallmark symptom of various neuropsychiatric diseases that leads to poor mental health outcomes throughout one's life and predicts poor psychosocial functioning.

Moreover, another research utilized a randomized, double-blind, placebo-controlled study involving 60 medical students with already high cognitive functions, which showed that *B. monnerri* extract (Bacognize) (150 mg twice a day) given for 6 weeks significantly improved cognitive function along with a significant rise in serum calcium (*p* < 0.05) (still within standard range *i.e.*, 9–11 mg/dL). The cognitive performance was validated by examining various neuropsychological tests (logical memory test, digit span memory task, etc.). These detailed memory assessments will provide a better insight into subtle memory deficits. Their study finding revealed no change in the brain's attention and sensory-motor performance, indicating that BME reduces participants' distractibility but somewhat improves cognitive functions (110).

A pilot study was performed to assess the Brahmi as a memory enhancer and its safety and tolerability in elderly patients of either sex. Each individual received 250 mg of Brahmi tablet (b.i.d.) for 3 months. All patients showed a rise in cognitive fitness with no significant adverse effect (111). In a recent study, it was found that the administration of *B. monnieri* extract (300 mg bid) in 19 patients for 4 weeks proved effective in the treatment of anhedonia when compared with the controls (23 patients) who have treated with citalopram 40 mg (TAU) (112). Table 2 summarizes the stated clinical trials of BME in humans.

**Table 2:** Summary of various clinical studies.

**S. No**.	**Study design**	**Dosage of BME**	**Intervention**	**Clinical outcomes**	**References**
1.	46 healthy participants (males = 11; females = 35). Aged between 18 and 60 years.	300 mg/day	12 weeks	Significant improvement in speed of visual information processing and learning rate. Concentrations were noticed with a reduction in state anxiety.	(103)
2.	107 healthy volunteers. Aged between 18 and 60 years.	300 mg/day	90 days	Increases accuracy and memory consolidation	(104)
3.	76 participants. Aged between 40 and 65 years.	300 mg for persons under 90 kg, and 450 mg for persons over 90 kg	90 days	Significant reduction in the rate at which freshly acquired information is forgotten	(105)
4.	98 healthy adults. Aged over 55 years.	300 mg/day	12 weeks	Improvement in-memory performance and retention.	(106)
5.	60 healthy elderlies (males = 23; females = 37).	300 mg/day	12 weeks	Improvement in the working memory, attention, and cognitive processing.	(107)
6.	17 healthy volunteers.	320 mg BM and 640 mg of BM	-	Reduced stress and improved mood.	(108)
7.	100 volunteers (male children and adolescents).	160 mg/day 320 mg/day	16 weeks	Increased cognitive function.	(109)
8.	60 Medical Students.	150 mg (b.i.d.)	6 weeks	Cognitive enhancement.	(110)
9.	12 patients. Aged 18 and more.	250 mg (b.i.d.)	3 months	Effective for treatment of dementia.	(111)
10.	19 patients.	300 mg bid	4 weeks	Effective for managing anhedonia.	(112)

### Toxicity and safety concerns

Despite the increased demand for herbal formulations in the market, there are several issues related to their safety. The safety of herbal medicine stands at a high-water mark with a significant increase in global consumption. There is currently confusion and prejudice regarding the safety of the herbal medication. As a result, public awareness, objective comprehension, and neutral and fair interpretation are required. Moreover, caregivers must understand what the drug is, its use, and how it should be administered. Medications should be kept out of the reach of people with cognitive impairment. It is one of the most demanding tasks for scientists and researchers to investigate the efficacy, adverse effects, and serious contaminants from mixtures of herbal formulations. The most important reasons for herbal drug toxicity are improper identification of plant material, contamination of herbs with toxins, pesticides, and heavy metals, and their interaction with conventional drugs upon concomitant intake. If strict standardization and quality control parameters have not been followed, the errors, including contamination of heavy metals, and excessive alcohol generation in formulations, lead to reverse effects (113).

**Figure 4:**
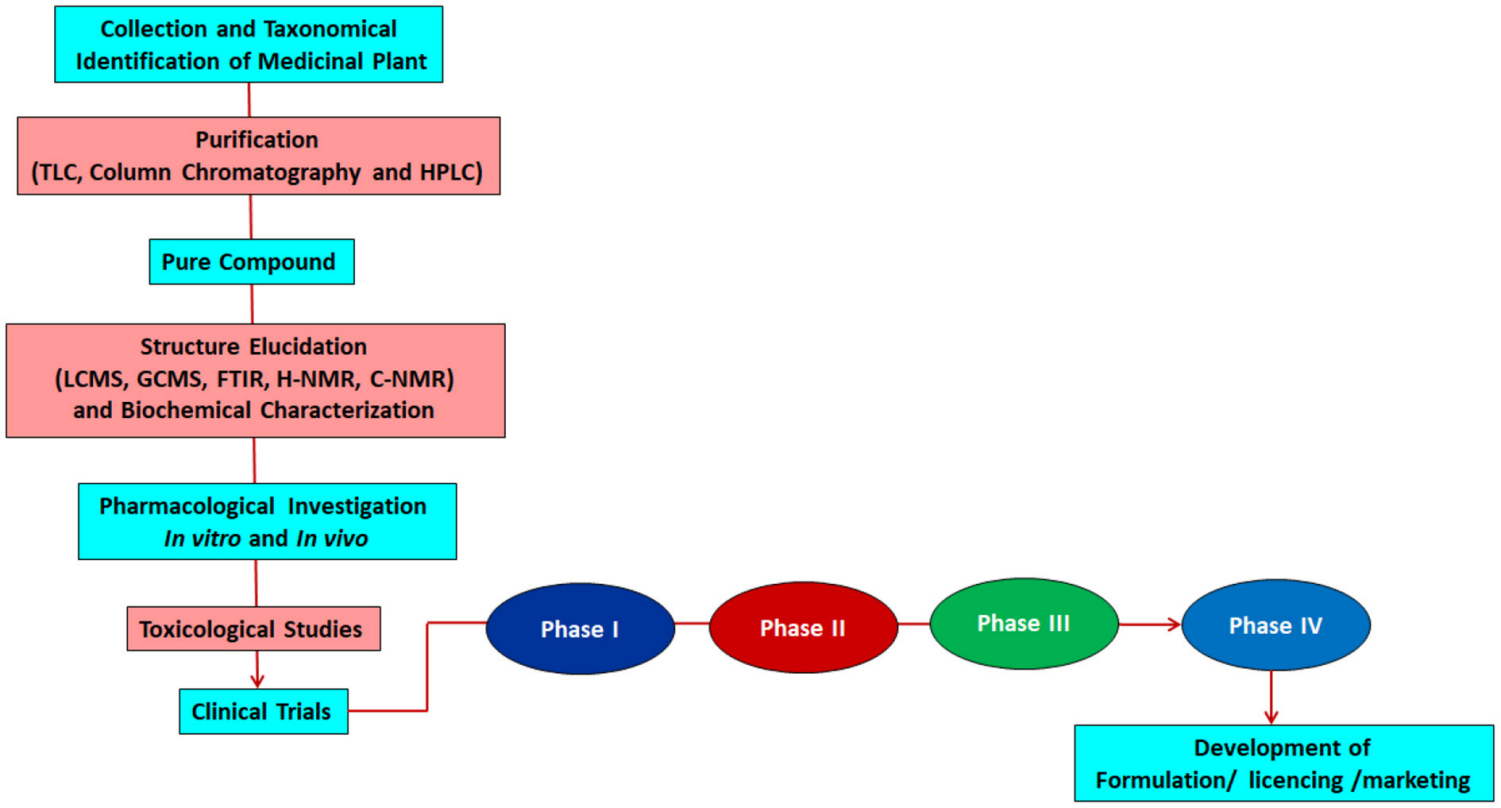
The Global concept of standardization, quality evaluation and pharmacological validation for the development of herbal medicine.

However, the herbal formulation can boost pharmaceuticals' effect and reduce the dosage of susceptible individuals. Even the most potent poison can become the best drug (114). Thus, there should be more focus on improving these herbs' bioavailability with the generation of minimum side effects. Quality control should be applied throughout the various processing stages, from the raw material to the finished product. A flow chart given in Figure 4 shows the standardized protocol for developing herbal formulations.

Several studies have evaluated the effectiveness of BME in an intoxicated animal model. In Sodium fluoride (NaF) intoxicated Swiss albino female mice, 300 mg/kg of BME reversed the effects of fluoride and impeded neuropathological alterations by restoring the cholinergic system and by decreasing the oxidative stress (115).

In 30% alcohol plus carbon tetrachloride (CCl4), intoxication of the Wistar Albino rats leads to hepatic oxidative stress that has been reversed by administering 100 mg and 200 mg/kg body weight/day of 70% ethanol extract of BM (116). Some studies revealed that 40 mg/kg of mBME can manage morphine-related hepatotoxicity and nephrotoxicity (117, 118).

Paraquat (PQ) exposure causes increased oxidative stress and mitochondrial dysfunction, followed by apoptosis and cell death. A standardized extract of *B. monnieri* neutralizes the PQ-mediated toxicity in *Drosophila* by optimizing oxidative stress, restoring ATP levels and decreasing apoptosis *via* inhibition of active JNK and cleaved Caspase-3 (119). Heavy metal toxicities have been recognized as a major public health risk. It interferes with the functions of various organs (liver, kidney) and systems (CNS, hematopoietic system).

Heavy metals cause oxidative stress and enzyme inhibition by interacting with the function of essential cations. Their accumulation ultimately leads to intellectual and behavioral impairments. Several studies in an *in vivo* model revealed the mitigating effect of BME on lead and aluminum-induced oxidative stress (120, 121). The methylated form of mercury (Hg), known as methylmercury (MeHg), is a ubiquitous environmental pollutant. MeHg-exposed rodents undergo oxidative stress in the cerebellum region leading to a deficit in motor performance.

The administration of Brahmi at 250 mg/mL concentration ameliorates the MeHg-Induced oxidative impairments (122). Consequently, BME has been proven promising in traditional medicine to protect the brain from oxidative damage resulting from heavy metal toxicity. The transcription factor nuclear factor-2 erythroid-related factor-2 (Nrf2) regulates the production of antioxidant genes *via* endogenous antioxidant (GSH level) mechanisms while also acting anti-inflammatory. Okadaic acid (OKA) administration resulted in memory impairment, decreased Nrf2 levels and caused oxidative stress and neuroinflammation in rats. Oral administration of BM (40 and 80 mg/kg) and melatonin (20 mg/kg) restored Nrf2 levels, decreased oxidative stress, and strengthened endogenous antioxidants. Thus, Nrf2 level modulation improved rats' spatial learning in the Morris water maze (123). *B. monnieri was* also found to attenuate trimethyltin (TMT)-induced cognitive impairments in mice by protecting the cholinergic system that promotes neurodegeneration in the dentate gyrus regions and protects the hippocampal neuron (124).

A study was conducted using wild type *C. elegans* model to evaluate the effect of BM and hexane extract (but not the ethanol extract) on glutamate exposure-induced AD parameters like mitochondrial stress and ROS production, as well as to assess the effects of hexane extract on the aging and life span of the model organism. Administration of 10 mg/ml *B.monnieri* hexane extract using ANOVA followed by Dunnett's *post-hoc* test showed a reduction in mitochondrial stress (*P* < 0.05) and ROS production (*P* < 0.0001) in cultured neuronal cells. Also, *B. monnieri* hexane extract at a dose of 7 and 10 mg/ml could extend the median and maximum lifespan and reduce the effects of aging in aged worms, thus, proving that BM hexane extract can be a potential prophylaxis agent against oxidative and mitochondrial damage and can be used as a therapeutic agent in aged patients (125).

Inappropriately, B. monnieri extracts did not induce toxicity or other symptoms in both female and male acute and chronic oral toxicity testing (126). Based on the results described above, we can conclude that *B. monnieri* is non-toxic and have the efficiency to reverse the adverse effect caused by toxic agents. Hence, *B. monnieri* is reliably safe for use for pharmacological purposes. However, more in-depth analyses are still required to explore the toxicity of the herb for human health-promoting benefits. The cited toxicity and prevention findings are summarized in Table 3.

**Table 3:** Efficacy of *B. monnieri* to attenuate the adverse effect caused by various toxic agents.

**S. No**.	**Toxicity induced**	**Dosage of BME**	**Findings with BME**	**References**
1.	Sodium Fluoride	300 mg/kg	Ameliorate the cholinergic system Attenuate the oxidative stress. Inhibited neuropathological alterations.	(115)
2.	30% Alcohol + CCL4	200/kg body weight	Protected the hepatic cells.	(116)
3.	Opioid	40mg/kg	Restored serum ALT, AST, and creatinine elevations. Shielded the liver and kidneys from the toxicological impact.	(117, 118)
4.	Paraquat	0.1% of BME (~50% of Bacosides) containing 20 mM PQ	Inhibit jnk2 mediated apoptosis through improved mitochondrial function and redox stabilization.	(119)
5.	Lead	10 mg/kg body weight/day	Reduced brain lead level when compared to conventional therapy. Attenuate the oxidative stress.	(121)
6.	Aluminum	40 mg/kg/day	Protect brain from oxidative damage.	(120)
7.	Methyl Mercury	250 mg/mL	Prevented mitochondrial damage. Attenuate the oxidative stress.	(122)
8.	Okadaic Acid	BM-40 and 80 mg/kg and Melatonin 20 mg/kg	The activation of Nrf2 and inhibition of NF-κB transcription factors by BME. Melatonin strengthens endogenous defense and protection against OKA induced memory deficit in rats.	(123)
9.	Trimethyltin	50 mg/kg	Ameliorates TMT-induced cognition dysfunction mainly via protecting the hippocampal neurons. They are promoting neuro-regeneration in the dentate gyrus regions.	(124)
10.	Glutamate	5 mM	Prevent mitochondrial damage. Prevent oxidative stress in cultured neuronal cells.	(125)

### Conclusion and future prospects

*Bacopa monnieri* extracts are extensively used to enhance memory and intelligence in Ayurvedic and Unani medicine systems. Extracts isolated from *B.monnieri* such as flavonoids, saponins, and triterpenes prevent oxidative and mitochondrial/ER stress and increase the aging duration in *C. elegans*. The present review summarizes recent findings on the potential health benefits of *B. monnieri*. Extracts of *Bacopa monnieri* such as Bacoside A, Bacoside B, Bacosaponins, and Betulinic acid play significant role in neuroprotection. The neuroprotective properties of these bioactive components include reduction of ROS, neuroinflammation, aggregation inhibition of amyloid-β and improvement of cognitive and learning behavior. Major phytoconstituents of *B. monnieri* are saponins such as bacoside A3, bacopaside II, X and bacopasaponin C and its isomer. Several authors reported inhibitory effects of bacoside on the glioma cell's viability and proliferation, indicating promising anti-cancer activity for the treatment of glioblastoma. Finally, we conclude that *B. monnieri* extracts could be implicated in treating Alzheimer's disease and other neurological disorders. However, future investigations are required to compare the neuroprotective effect of *B. monnieri* extracts with standard drugs to establish systematic clinical uses.

## Author contributions

Conceptualization and writing—review and editing: UF and MH. Methodology: UF and SR. Software and resources: SAh and SAl. Validation: WE, IK, and RA. Formal analysis: MA. Investigation: AI. Data curation: SR. Writing—original draft preparation: UF. Visualization: SAh. Supervision: SAl and IK. Project administration: WE. Funding acquisition: MH. All authors have read and agreed to the published version of the manuscript. All authors contributed to the article and approved the submitted version.

## Funding

This work was supported by the National Medicinal Plants Board (NMPB) and Ministry of AYUSH, Government of India [Z. 18017/187/CSS/R&D/DL-01/2019-20-NMPB-IVA].

## Conflict of interest

The authors declare that the research was conducted in the absence of any commercial or financial relationships that could be construed as a potential conflict of interest.

## Publisher's note

All claims expressed in this article are solely those of the authors and do not necessarily represent those of their affiliated organizations, or those of the publisher, the editors and the reviewers. Any product that may be evaluated in this article, or claim that may be made by its manufacturer, is not guaranteed or endorsed by the publisher.
